# Evidence on language monitoring in primary healthcare: a scoping review

**DOI:** 10.1590/2317-1782/e20240189en

**Published:** 2025-07-14

**Authors:** Ivana Vieira de Resende Teixeira, Ana Carolina Andrade Valadares, Stela Maris Aguiar Lemos

**Affiliations:** 1 Programa de Pós-graduação em Neurociências, Universidade Federal de Minas Gerais – UFMG - Belo Horizonte (MG), Brasil.; 2 Programa de Pós-graduação em Ciências Fonoaudiológicas, Universidade Federal de Minas Gerais – UFMG - Belo Horizonte (MG), Brasil.; 3 Departamento de Ciências Fonoaudiológicas, Universidade Federal de Minas Gerais – UFMG - Belo Horizonte (MG), Brasil.

**Keywords:** Language Development, Public Health Surveillance, Primary Health Care, Child, American Speech-Language-Hearing Association

## Abstract

**Purpose:**

To map scientific productions on language monitoring in primary healthcare.

**Methods:**

This scoping review was guided by the following research question “What is the evidence of language development in early childhood in primary healthcare surveillance?”. Studies were searched from February to November 2023 in the MEDLINE, LILACS,Cochrane, Scopus, Web of Science, and EMBASE databases. It included original research articles and reviews with no restriction on time or language and excluded samples with suspected or confirmed diagnoses of intellectual disability, hearing loss, and global developmental delays. Two researchers independently screened titles and abstracts and read the full text of the articles. References were managed in Rayyan.

**Results:**

The search found 436 studies, of which 220 were excluded for being duplicates and 177 for not answering the research question. Hence, 39 articles were read in full text, of which 31 were excluded for not addressing the topic. Eight articles were included in the review (six original research articles and two reviews). The studies collected data from children aged 1 month to 5 years who visited primary care units for vaccinations or consultations, and during home visits. The use of instruments and clinical observation were the strategies used to investigate language development. The prevalence of language delay ranged from 2.5 to 31%.

**Conclusion:**

Language development is not yet regularly monitored in child health surveillance in primary care.

## INTRODUCTION

Language is an important domain of neuropsychomotor development that encompasses the phonetic-phonological, semantic-lexical, morphosyntactic, and pragmatic subsystems^(1-3)^. It develops in the first years of life and can be considered a predictor of the child's performance in later stages^(1)^.

Due to the combination of genetic, biological, and environmental factors, language is the domain of child development most susceptible to delays^(4)^. Language delays comprise the delayed development of age-expected language skills when other justifiable causes are absent^(5,6)^. Delays are often observed in high-risk populations, such as those at economic disadvantage^(1)^.

Children with language delays are at greater risk of adverse outcomes in childhood and adulthood, such as social isolation, mental health, literacy, and employability problems, and low income^(6,7)^. Moreover, language delays may also indicate more serious and persistent neurodevelopmental problems^(2)^. Therefore, timely identification and intervention can prevent negative outcomes and improve children's quality of life^(5)^.

The increased prevalence of changes in child development, including language delays, has been identified as a public health problem^(2)^. Monitoring child development has been identified as a key element of primary care practices^(8)^. The Healthy Child Programme (HCP), implemented by the NHS, is a notable example of a public policy that offers periodic assessments from prenatal care to the early years of life to identify developmental delays and implement appropriate interventions in a timely manner^(8,9)^. Studies indicate that programs like the HCP promote better cognitive and social outcomes, particularly when combined with robust and integrated public policies^(9-12)^. In addition, global initiatives such as the WHO-UNICEF Care for Child Development demonstrate that interventions carried out by primary care professionals are effective in promoting child development, even in contexts of higher socioeconomic vulnerability^(13)^. Systematic reviews reinforce that these programs, when combined with strategic public policies, significantly contribute to equity and comprehensiveness in early childhood care^(13)^. Thus, monitoring child development in primary healthcare (PHC) is essential to enable and implement developmental surveillance strategies^(5,14)^. Surveillance has been recommended for more than 27 years and is a flexible, longitudinal, and ongoing process in which healthcare professionals monitor and identify children who may have developmental delays^(15-17)^. Although the studies are not specifically in the field of language, they emphasize the importance of implementing strategies that adapt to the territorial, economic, and cultural specificities to ensure that all children have the opportunity to reach their full development, including language development. However, the literature shows discrepancies regarding the feasibility of identifying language delays in primary care^(2,18)^. There are considerable, insufficiently described gaps in the literature regarding the methods to monitor and strategies to follow up on linguistic development in the early years. Hence, this study aimed to map scientific productions on language monitoring in PHC.

## METHODS

This scoping review followed the JBI Manual for Evidence Synthesis and was reported with the guidelines of the Preferred Items for Systematic Reviews and Meta-analysis – Extension for Scoping Reviews (PRISMA-ScR)^(19)^. The review sought to answer the following research question: “What is the evidence of language development in early childhood in PHC surveillance?”. The PCC mnemonic (Population, Concept, and Context) guided the search for studies, namely: (a) population: preschoolers; (b) concept: language development in public health surveillance; (c) context: PHC. The review was registered on OSF Support.

### Search strategy

The strategy used the following descriptors (Medical Subject Headings [MeSH] and Health Sciences Descriptors [DeCS]): “Infant”, “Children”, “Child, Preschool”, “Language Development”, “Public Health Surveillance”, “Child Care”, “Primary Health Care”, and their respective terms in English and Spanish. The descriptors were combined with Boolean operators to formulate the search equation, considering the language of each database (Table 1). The search was conducted in the databases of MEDLINE, via the US National Library of Medicine (PUBMED), LILACS, via the Virtual Health Library (VHL), Cochrane, Scopus, Web of Science, and EMBASE. Data were collected from February to November 2023.

**Table 1:** Database search strategies

Database	Search strategy
Lilacs	(“Desenvolvimento da Linguagem” OR “Language Development” OR “Desarrollo del Lenguaje” OR “Développement du langage oral” OR “Aquisição da Linguagem” OR “Linguagem Infantil” OR “Child Language” OR “Lenguaje Infantil” OR “Langage de l'enfant” OR “Linguagem da Criança” OR “Language Acquisition”) AND (“Atenção Primária à Saúde” OR “Primary Health Care” OR “Atención Primaria de Salud” OR “Soins de santé primaires” OR “Vigilância em Saúde Pública” OR “Public Health Surveillance” OR “Vigilancia en Salud Pública” OR “Surveillance de la santé publique” OR “Plano de Vigilância em Saúde” OR “Vigilância da Saúde” OR “Vigilância da Saúde Pública” OR “Vigilância em Saúde”)
MEDLINE via PubMed	(“Language Development” OR “Child Language” OR “Language Acquisition”) AND (“Primary Health Care” OR “Public Health Surveillance”)
Cochrane	
Scopus	
Web of Science	
EMBASE	('language development' or 'child language' or 'language acquisition') and ('primary health care' or 'public health surveillance')

### Selection criteria

The review included original research articles and reviews that answered the research question with no restrictions on time or language. The exclusion criteria were samples with a suspected or confirmed diagnosis of intellectual disability, hearing loss, and global developmental delay.

### Selection process

All studies identified in the screening phase were included and managed at Rayyan Qatar Computing Research Institute ^(20)^. Two researchers independently screened and removed duplicates, initially screened studies based on their titles and abstracts, and read the full text of screened articles, following the eligibility criteria. Disagreements were resolved by consensus involving the other authors.

### Data extraction and synthesis

The following data were extracted from the selected studies: study design, participant characteristics, collection strategies and instruments, and main outcomes (Table 2).

**Table 2:** Overview of the studies | Monitoring and language development in primary healthcare (PHC)

Authors	Study design	Country/ Period	Sample characteristics					
			Total N	Age	Assessment characteristics	Instruments	Aspects assessed	Main outcomes
Frelinger et al. (2023)^(21)^	Retrospective	United States of America	14.559	12-60 months	Nosological diagnosis	ICD-10^1^	ASQ^2^ or clinical observation	14.2% language and speech delays.
Alakeely et al. (2022)^(5)^	Cross-sectional	Saudi Arabia	250	12-60 months	Clinical assessment and self-administered questionnaire for parents/guardians	Questionnaire for parents/guardians and the Arabic Language Milestone Screening Test	Language development milestones	22.2 to 31% prevalence of language delay among those 1 to 5 years old. The absence of breastfeeding was related to the likelihood of language delays (p = 0.014).
Santos-Alvarez et al. (2021)^(25)^	Cross-sectional	Mexico March 2018 to November 2019	69	22-59 months	Clinical observation and questionnaire for parents/guardians	EDI^3^	Gross motor Fine motor Social Language Learning	Developmental delay: 13%. Risk of delay: 16%. Language was the domain with the highest risk of delay (p = 0.00).
Wagner et al. (2017)^(24)^	Prospective longitudinal	Brazil March to August 2015	25	1-12 months	Questionnaire for parents/guardians	Questionnaire to monitor hearing and language in the first year of life	Hearing and language milestones	4% of the sample had RFHL^4^ and 4% had to be referred for EOA^5^ retest in the absence of RFHL.
van der Linde et al. (2016)^(22)^	Cross-sectional	South Africa	201	6-12 months	Clinical assessment	RITLS^6^	Pragmatics Gestures Play Language comprehension and expression Interaction-attachment	The study identified 13% of language delays, of which 58% had a delay in one communication domain and 4% in five domains. Expressive language was the domain with the greatest delay (84.6%). Having more than three siblings (p = 0.054), informal housing or staying with other people (p = 0.024), and maternal age under 18 or over 35 years old (p = 0.035) were significantly associated with language delays.
Hannus et al. (2009)^(23)^	Retrospective	Finland 1989 to 1999	2.480	0-180 months	Nosological diagnosis	ICD-9 and ICD-10^1^	Language comprehension and expression	2.5% prevalence of language development delay and language disorder.

1 International Statistical Classification of Diseases and Related Health Problems

2 Ages and Stages Questionnaire

3 Evaluación del Desarrollo Infantil

4 Risk factors

5 Evoked Otoacoustic Emissions

6 Rossetti Infant-Toddler Language Scale

## RESULTS

**Figure 1:**
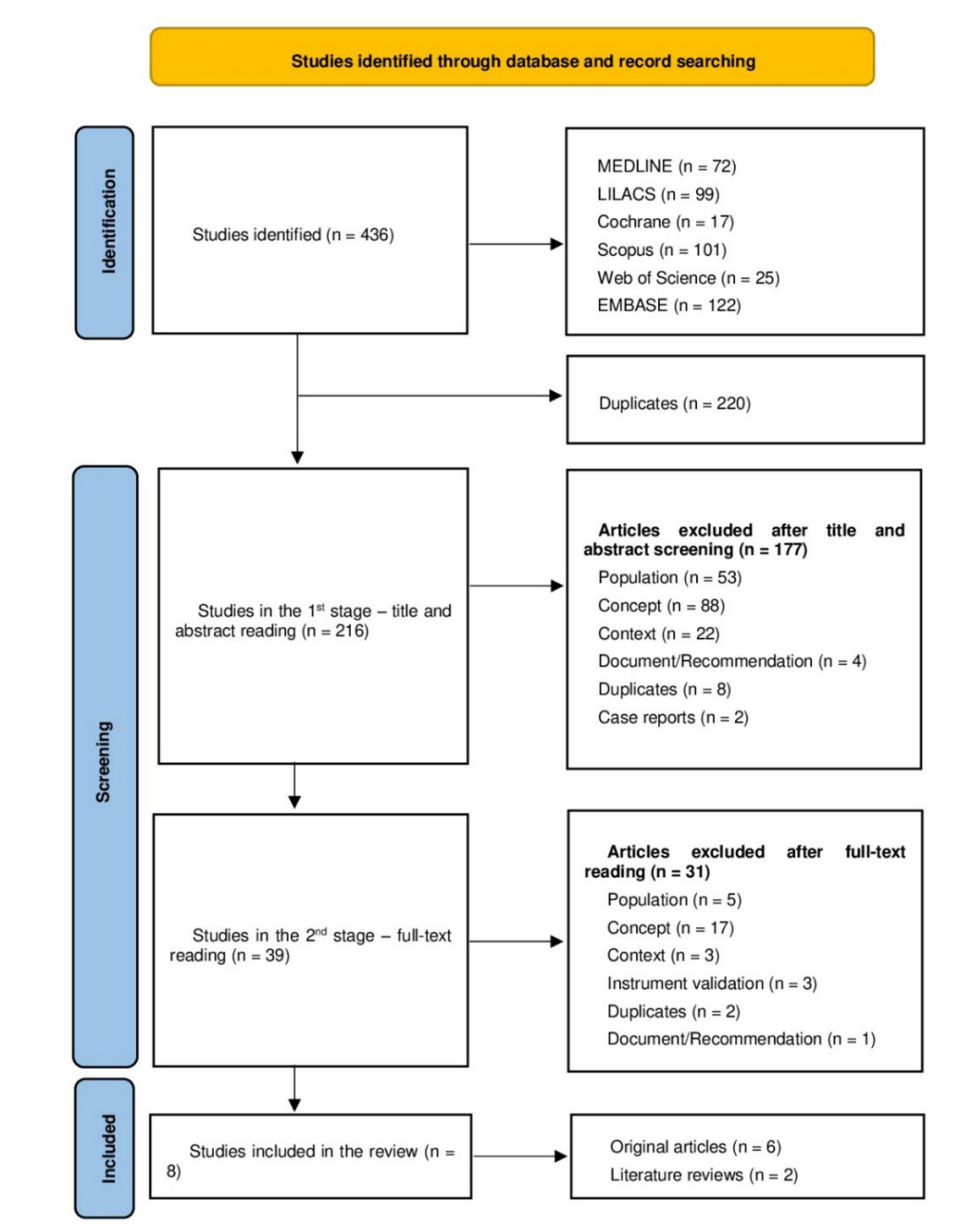
PRISMA 2020 flowchart with the study selection process

The search located 436 studies, of which 220 were removed for being duplicates and 177 for not answering the research question after reading their titles and abstracts. Hence, 39 articles were read in full, and 31 were excluded for not addressing the topic (Figure 1). Of the eight articles that met the inclusion criteria, six are original research (Table 2) and two are literature reviews (Table 3).

**Table 3:** Overview of the studies | Literature reviews

Authors	Study design	Country/ Period	Database	Total N	Main outcomes
Jullien (2021)^(6)^	Scoping review	Germany	Cochrane	11 reviews, laws, and protocols	The screening tools used to assess speech and language delay in primary healthcare had a wide range of accuracy.
		November 2019			
Nelson et al. (2006)^(3)^	Systematic review	United States of America	MEDLINE, PsycINFO, and CINAHL	87 articles	There was an absence of gold-standard instruments to screen speech and language delay.
		November 2004			

The overview of the studies is detailed in Table 2. Most of the six original research studies collected data from children aged 1 month to 5 years who visited PHC units for vaccination or consultation (n = 4)^(5, 21-23)^ and during home visits (n = 1)^(24)^. The general characteristic of the studies was instruments or clinical observation to investigate the development of language and other skills expected for their age, used by speech-language-hearing pathologists (n = 2)^(22,23)^, pediatricians (n = 1)^(21)^, nurses (n = 1)^(5)^, and community health workers (n = 1)^(24)^. One of the studies did not report which professionals collected data ^(25)^.

The studies that assessed language development in PHC were carried out in the United States of America^(21)^, Saudi Arabia^(5)^, Mexico^(25)^, Brazil^(24)^, South Africa^(22)^, and Finland^(23)^. The method used in these studies to monitor language development was a combination of questionnaires administered to parents/guardians and the direct observation of language (n = 2)^(5,25)^, direct observation alone (n = 2)^(22,23)^, or only questionnaires for parents/guardians (n = 1) ^(21)^. The study by Frelinger et al 2023 reported a varied use of validated questionnaires and the professionals’ clinical experience, according to data described in the medical records.

The prevalence of language delay ranged from 2.5 to 31%^(5,21-23,25)^. The absence of breastfeeding, the number of siblings, housing condition, maternal age, and the child's sex were identified as risk factors associated with the identified delays (Table 2)^(5,21-23)^.

The two literature reviews that met the criteria of the present one approached the use of instruments to screen speech and language delays in PHC, their accuracy, adverse screening effects, and evidence of intervention (Table 3)^(3,6)^.

## DISCUSSION

Evidence from language monitoring in PHC points to an increased prevalence of identified language delays^(5,21-23,25)^, although monitoring is not yet universally practiced in child health surveillance^(18)^.

A prevalence of 2.5 to 31% of language delays was observed in PHC^(5, 21-23,25)^, and language was the domain with the highest prevalence of delays in preschoolers^(4)^. This prevalence can reach 59% when compared with motor delays in children undergoing timely intervention. Delays may persist in 40 to 60% of cases when there is no intervention, given their harmfulness in childhood and adulthood, with academic, social, attentional, employability, and low-income consequences^(3,4)^. This makes language disorders one of the most prevalent developmental disorders^(2,21)^. Considering it along with the other child developmental changes, which have been identified as a public health problem^(2)^, joint and interprofessional actions must be implemented to identify and intervene in the area where these populations live^(4)^. The heterogeneity in the prevalence rates of delays can be attributed to the accuracy of the screening instruments used, diagnostic criteria, the trajectory of language development, sociocultural factors, and the ability of healthcare professionals to identify delays^(26)^_._

Language development may be limited by biological factors and unfavorable psychosocial environments^(27,28)^. A limited portrayal of such aspects is found in the literature, reflecting the scarcity of studies that address social health determinants related to language development. Developmental assessments commonly do not address information about the children's context, even though it is essential to understand the environment and the family^(1)^. A broader approach to understanding these factors is essential to better comprehend language acquisition in the population due to its biopsychosocial nature and the extent of the impacts of language delays.

Males predominated in the studies and the identification of delays in this population^(3,5,21-25)^. Being a male, which has been pointed out as a risk factor for language deficits, had controversial results in studies included in a systematic review, suggesting possible bias in the inclusion of clinical samples for being more frequently referred to health services for reasons that are not yet clear^(26)^.

The studies identified pre-, peri-, and post-natal biological risk factors, including bleeding during pregnancy, urinary infections, cervicovaginitis, maternal hypertension, maternal age below 18 and above 35 years, absence of breastfeeding, admission to the neonatal ICU, and having more than three siblings^(5, 22-25)^. The literature reports a series of other risk factors identified in children monitored in PHC, such as prematurity, low birth weight, gestational age, use of mechanical ventilation, low Apgar scores, and respiratory distress^(27)^. These risk factors were associated with language development and negatively influenced it. Identifying and monitoring these factors can provide follow-up actions for children at risk through mother-and-child public health policies.

The parents’/guardians’ education was the only psychosocial factor mentioned in the studies, ranging from elementary school to bachelor’s degree. The mothers reached higher education levels, which is potentially important due to their influence on child development^(7)^. Children whose mothers only reached elementary education or did not have equivalent education are already at a cognitive and language disadvantage when they enter school, with lower academic performance, contributing to the intergenerational transmission of social inequalities. The prevalence of language difficulties is higher in economically disadvantaged communities than in the population as a whole. Therefore, such aspects should be considered in population studies in PHC.

Although the identification of delays has been increasingly recommended to provide timely intervention^(4,15)^, its operationalization faces many challenges, mainly how delays are identified. The studies used self-administered questionnaires for parents/guardians, questionnaires administered by health professionals, and clinical language assessments^(5, 21-25)^. The accuracy of parent/guardian self-reported instruments did not differ from those administered by professionals in the literature, proving them to be a viable and low-cost option^(18)^.

The instruments used in the studies had different local validation criteria, concepts, and language assessment methods. The Ages and Stages Questionnaire (ASQ) is a screening instrument based on guardians’ reports, whose questions address social, gross motor, fine motor, problem-solving, and communication domains^(21, 29)^. The ASQ, which has been validated and adapted for several countries, is an easy-to-apply and low-cost overall screening scale. The Arabic Language Milestone Screening Test is a self-administered questionnaire validated by the Centers for Disease Control and Prevention (CDC) and addresses sociodemographic aspects and milestones in the development of the Arabic language^(5)^. The *Evaluación del Desarrollo Infantil* (EDI, Child Development Evaluation) is a questionnaire that screens global developmental problems, developed and validated in Mexico, addressing risk factors, warning signs, areas of development (fine motor, gross motor, language, social, and knowledge), warning signs, and neurological examination^(25, 30)^. The Questionnaire for Monitoring Hearing and Language Development in the first year of life, validated in Brazil for application by community health workers, has three questions – one related to the parents' perception of children's hearing and two on language development milestones up to 12 months old^(24)^. The Rossetti Infant-Toddler Language Scale (RITLS) is a comprehensive and easy-to-apply tool validated to assess verbal and pre-verbal communicative skills and interaction of babies and young children in terms of pragmatics, gestures, play, language comprehension and expression, and interaction-attachment^(22)^. Also, the Modified Checklist for Autism in Toddlers (M-CHAT) was routinely used in consultations in San Francisco to screen autism spectrum disorder in 18-to-24-month-old children^(21)^.

Development surveillance must prioritize the use of standardized instruments because informal observations and checklists are unable to identify everyone who has developmental delays^(14,16)^. Screening can help identify and classify the severity of delays and define the need for follow-up^(16)^. Furthermore, its use is recommended due to its quick and easy application, especially in public health, and the widely documented evidence of its accuracy in the literature^(14)^. Given the unstable trajectory of language development in the first years of life, it is essential to use instruments that predict long-term language outcomes in the continuous surveillance of all children’s development, regardless of risk^(31)^, which must take place in every childcare consultation^(16)^ because language delays may indicate other neurodevelopmental disorders, such as global developmental delay, autism spectrum disorder, and hearing loss^(2,21,31)^.

Strategy variability is a common difficulty in low- and middle-income countries due to the scarcity of validated instruments^(14)^, and the lack of cross-culturally adapted instruments, useful for comprehensive PHC assessments in different countries, hinders population analyses of language monitoring^(3,6,18)^. Furthermore, the use of different types of instruments that assess different aspects of child development does not always address the language construct in all its subsystems (phonetic-phonological, semantic-lexical, morphosyntactic, and pragmatic). The limited number of instruments and the design, variability, and accuracy of screening tools may explain the absence of language monitoring in PHC^(3,5,18)^, which highlights the need to develop universal tools that allow comparisons between the most diverse contexts.

The lack of knowledge among PHC professionals regarding language acquisition and development was also reported in the studies as a barrier to development surveillance^(24,32)^, which makes it difficult to identify delays and provide assertive guidance to the population^(17,24,32)^. The composition of professionals working in Primary Health Care (PHC) can vary from country to country, usually consisting of doctors and nurses, and in developing countries, supported by multiprofessional teams. Access to care for communication disorders in PHC is still limited. In Brazil, only 21.8% of Family Health Strategy teams have a speech therapist, who works in the assessment and rehabilitation of communication and swallowing disorders^(33)^_._ Thus, it is essential to provide training for the entire team and its managers, emphasizing the need for continuous education of the professionals working in Primary Health Care (PHC).

The studies included in the review did not show whether language monitoring surveillance is a routine practice in primary care in the countries where they were conducted. However, child development surveillance has been recommended, and several instruments reported in the literature include aspects related to language^(14)^. Furthermore, initiatives such as Nurturing Care support that parental programs addressing nutrition and stimulation have been effective in improving cognitive and language development, covering key aspects of early childhood development care included in the United Nations Sustainable Development Goals (UN)^(34)^.

The limitations of the review include the scarcity of validated universal instruments, which prevent in-depth population analyses of language development in the most diverse contexts, and the lack of adequate training of health professionals. Moreover, the studies did not show how public health policies have been incorporated into primary care to ensure the monitoring and promotion of full linguistic development.

Regarding advances, the present review addressed the evidence on language monitoring in PHC and its operationalization, although the scope of the information still has limitations and gaps. The results of the review suggest preliminary evidence about the possibility and need for timely identification of language development delays in PHC to guide local intervention strategies and public policies, such as enabling the training of professionals working in primary care and increasing the participation of speech therapists in PHC.

Thus, further studies should be carried out to develop universal screening tools that consider language in all its subsystems and address biological, social, and local aspects to assess the language development of children followed up in PHC, demonstrate the feasibility of language monitoring, and guide its operationalization. Furthermore, future studies should consider psychosocial factors such as parental education, socioeconomic classification, and race. Studies addressing strategies for implementing language monitoring and its effects in primary care are also necessary for a better understanding of effective health surveillance practices, considering local specificities and the potential to reduce inequalities in child development.

## CONCLUSION

The mapping showed that child health surveillance in PHC does not regularly monitor language development yet, although the literature has increasingly reported a greater prevalence of delays. Various biological and psychosocial factors impact language development, requiring a broader approach to understanding language development in the population due to its biopsychosocial nature.

Language monitoring in PHC should prioritize standardized and universal instruments thanks to their easy application, low cost, and accuracy in identifying language delays. To this end, the instruments must have good psychometric capabilities, address all language subsystems, and consider biopsychosocial factors. Continuing education for health professionals is also a strategy that can advance the process of identifying delays and providing assertive guidance to the population in PHC, ensuring comprehensive care for early childhood.
